# Oligodendrocyte: Development, Plasticity, Biological Functions, Diseases, and Therapeutic Targets

**DOI:** 10.1002/mco2.70618

**Published:** 2026-02-08

**Authors:** Qiong Xiang, Ruo‐Lan Shi, You‐Xia Huang, Li‐Ni Liu, Jia‐Sheng Tao, Xian‐Hui Li, Xiao‐Da Li

**Affiliations:** ^1^ Institute of Medicine Jishou University Jishou Hunan China; ^2^ Peking University Health Science Center Beijing China

**Keywords:** axonal plasticity, bioengineering technologies, central nervous system diseases, oligodendrocyte progenitor cells, oligodendrocytes

## Abstract

In the past few years, the incidence rate of central nervous system (CNS) diseases is still growing. Meanwhile, the molecular mechanism on the pathogenesis of neurological diseases remains elusive. Oligodendrocyte progenitor cells (OPCs) are distributed in the whole CNS and represent a population of migrating and proliferating adult progenitor oligodendrocytes that can be differentiated into oligodendrocytes (OLs). The main function of OLs is to produce myelin, the membrane wrapping tightly around the axon, which are associated with the myelination and remyelination. During regeneration, the new OLs from OPCs can regenerate lost myelin, which prevents axonal degeneration and restores its plasticity and function. Considering these energy‐consuming processes, the high metabolic turnover OLs are susceptible to neurotoxic factors and its excitatory toxicity. Thus, the pathogenesis of OPC and OL are proven in neurological diseases, such as multiple sclerosis, Alzheimer's disease, major psychiatric diseases, and epilepsy. The current study reviewed the development, plasticity as well as application of OPCs and OLs researches on CNS diseases. Additionally, the effective methods and bioengineering technologies as well as biomaterials relevant to regenerative medicine are also discussed, which could provide the novel insight into the therapeutic treatment of those diseases, exploring new pathological clues, identifying the key molecules and targets as well as the potential biomarkers.

## Graphical abstract

The oligodendrocyte lineage and its roles in development, plasticity, and CNS homeostasis have been shown to have the potential to treat neurological diseases. However, the main obstacles to clinical translation, particularly in terms of BBB challenges and precise delivery. To date, the nanocarrier drug delivery system is one of the promising ways and efficiently applied in numerous CNS diseases.

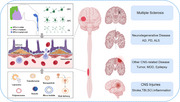

## 1 Introduction

Central nervous system diseases are currently one of the major global health issues [1]. Despite years of research on CNS diseases, they still face significant clinical challenges since complicatedly pathological mechanisms and limited diagnosis [2]. It is widely accepted that spatial organization, cell type transformation as well as variability of gene expression and also the signal cascades regulation and modulation are contributed to the developing process of the diseases. Oligodendrocyte progenitor cells (OPCs) as the neural stem cells (NSCs) are distributed in the whole CNS and represent a population of migrating and proliferating adult progenitor oligodendrocytes (OLs) that can be differentiated into OLs. Generally, the CNS exhibited its function with a distinctive way on the basis of regions or circuits involved, particularly in disease ecosystems with much higher frequent in cellular communications and interactions [3]. And now, many evidence suggest that OPCs are independent of their role as precursor cells of OLs, associated with brain plasticity through the integration of synaptic activity and long‐term enhanced mediation as well as the appearance of depressive behavior [4, 5]. Additionally, it has been investigated and applied to the treatment of several types of disease, including spinal cord injuries, Alzheimer's disease (AD), Parkinson's disease (PD), stroke, multiple sclerosis (MS), and so on. To date, stem cell therapy has been widely used in clinical trials. Moreover, mesenchymal stem cell (MSC) transplantation has already been treated routinely for diseases in many countries [6]. Similar to MSCs, with OPCs, confident evidences have presented wide range of treatments for immune regulation and tissue regeneration; however, the molecular mechanism in neurological diseases remains elusive. Meanwhile, in neurodegenerative diseases, the decline in the function of stem cells and progenitor cell populations is a critical reason for the decrease in tissue regeneration capacity, among which OPCs are mainly affected. There was evidence that suggested that the OPC microenvironment determines the aging process of OPCs. For instance, transferring senescent OPCs into the stem cell pool of young animals can significantly improve the function of OPCs, indicating that the microenvironment influences cellular senescence. Consistent with this, young cerebrospinal fluid restores OL formation and long‐term memory consolidation in aged mice. These highlight the significance of reducing OPC aging in improving the progression of neurodegenerative diseases and the potential mechanisms include promoting OPC maturation and enhancing myelin regeneration. The OL lineage and its fundamental biological roles in development, plasticity, and CNS homeostasis have been shown to have the potential to treat CNS diseases. However, the main obstacles to clinical translation particularly in terms of precise delivery, low cell survival rates, and blood‐brain barrier challenges.

Currently, drug delivery system is a promising tool that possesses fine physicochemical properties and can enhance drug delivery efficiency by improving adverse drug characteristics, increasing permeability, improving tissue distribution, and in vivo metabolism. It has been widely used in numerous diseases, of course in CNS diseases [7, 8, 9, 10]. For instance, vehicle that effectively penetrates the BBB and delivers treatments to the brain [11]. In addition, oxidized tannic acid‐modified gold nanocrosslinkers were synthesized and effectively crosslinked with chitosan to prepare hydrogels, which were investigated in vitro to promote the proliferation and differentiation of NSCs [12]. And also, the potential applications of gold nanoparticles (AuNPs) in therapy of glioblastoma multiforme (GBM) which is an interested ongoing field of research in recent years. [13]. Meanwhile, the ionizable lipid nanoparticles (LNPs) combined with mRNA delivery into the perinatal brain may supply a translatable platform in clinic for mRNA therapy as well as gene editing [14]. However, the stability of these and other nanoparticles in the circulation need to be considered. In fact, the PEGylation of nanomaterials was successfully used to prolong the circulation of nanoparticles. Therefore, the multifunctional nanocarrier for the treatment of AD was administered intranasally to achieve the codelivery of small interfering RNA and rapamycin into the brain [15]. However, Li et al. have first discovered that subcutaneous injection of polylactic acid hydroxyacetate (PLGA) nanoparticles containing the peptide MOG35‐55 is sufficient to significantly improve symptoms in a mouse model of MS, which induce lower complement activation, neutrophil recruitment, and costimulatory molecule expression in dendritic cells, creating a more tolerant microenvironment in vivo [16]. Actually, targeting drugs to the brain is one of the most challenging problems as the BBB is unable to bring drugs to the brain [17]. Among many ways to overcome the limitations of the BBB, the nanocarrier drug delivery system/platform is one of the promising ways to deliver therapeutic drugs or imaging/diagnostic agents to the CNS, which can provide protection for therapeutic agents and efficiently deliver drugs to specific sites and reduce systemic drug toxicity [18, 19, 20]. Considering OPCs as a type of stem cell and its multifunctionality, combining them with nanomaterials is an encouraging approach that can promote effective delivery and functional effects of therapeutic agents [21]. In addition, cell membrane‐coated nanocarrier‐based delivery systems are also designed to exhibit a broad range of features, such as potential immunogenicity, prolonged circulation time, and specific targeting [22, 23, 24]. Therefore, the biotechnology strategy of synthesizing nanocarriers combined with cell membranes for enhancing cell therapeutic efficacy has received much attention in recent years [25].

Here, we systematically reviewed the development, plasticity, and biological functions of OL lineage, while critically evaluating how advanced delivery strategies can unlock its therapeutic potential across CNS diseases. And then, we finally discussed possible directions for making progress in this field.

## 2 Development, Plasticity, and Biological Functions of OL Lineage

### 2.1 The Development of OL Lineage

**FIGURE 1:**
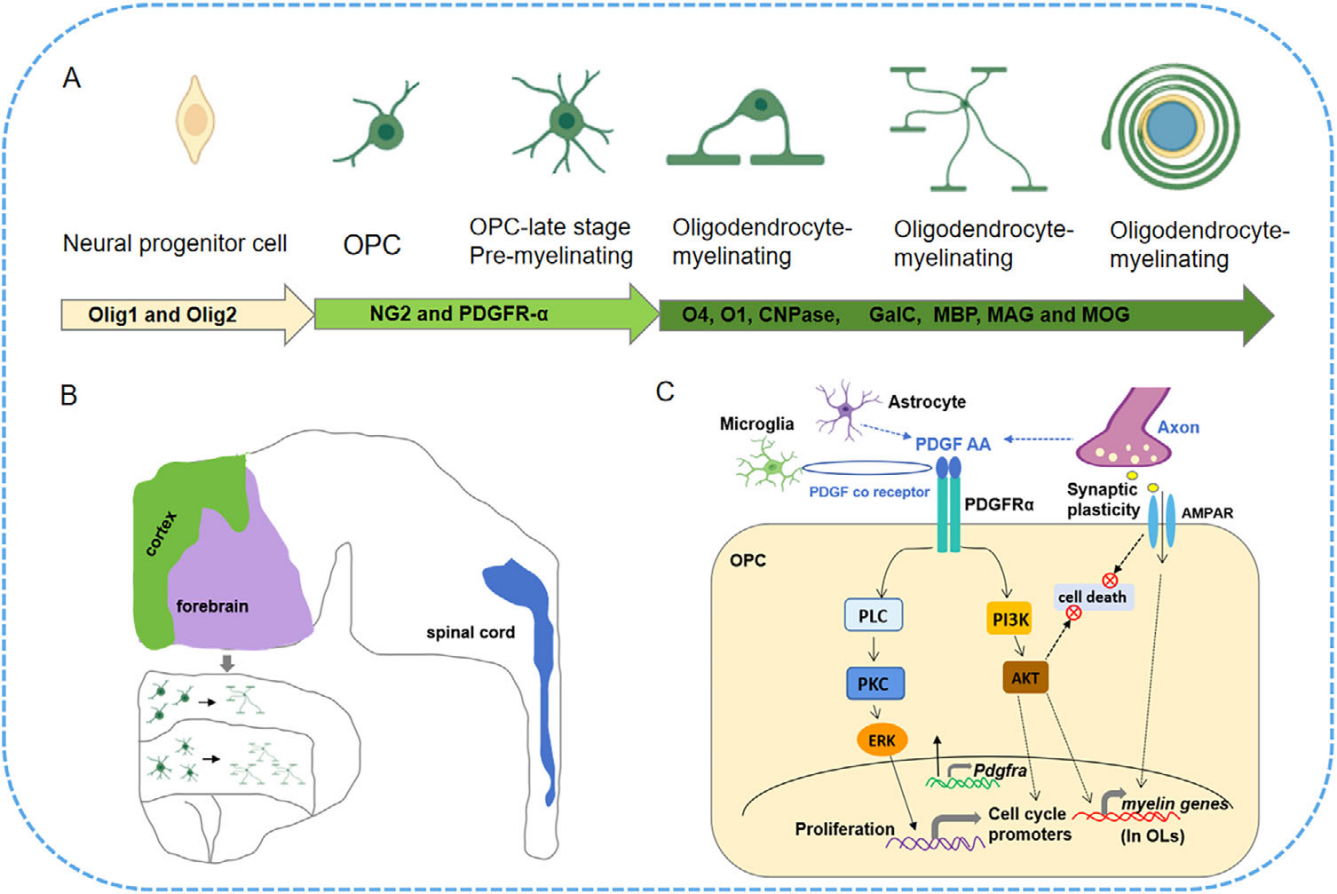
The development and intracellular signaling pathway of OPCs. (A) The developmental process with different markers in OPCs and OLs. Oligo1 and Oligo2 are the markers of the all cells of oligodendrocyte lineage, while OPC and premyelinating OLs are characterized by NG2 and PDGFR‐α expression. O4, O1, and CNPase are expressed on myelinating OLs, while axonal myelin OLs are characterized by GalC, MBP, MAG, and MOG. (B) OPCs in the white matter elongate, proliferate, and differentiate into OLs in forebrain and cortex. (C) OPC intracellular signaling pathway. PDGF‐AA combined with PDGFRα mainly activates ERK and Akt in OPCs, promoting their proliferation, survival, and differentiation, and activating myelin gene transcription during OL maturation. Besides, microglia and astrocytes can indirectly affect OPCs; in addition, neuronal activation may promote OL differentiation through AMPA receptors.

As known, OPCs are multipotent progenitor neural cells with self‐renewal and multilineage differentiation potential [26]. OPCs are distributed throughout the CNS and represent a group of adult progenitor cells that can differentiate into OLs through migration and proliferation [27] (Figure 1A). OLs are one of the types of glial cells in the CNS, in addition to microglia and astrocytes. In the glial cell population, bipolar cells have been found to have high proliferation and migration, while glial cells produced by filamentous myelin mainly exist in white matter (WM), showing a bipolar precursor cell type, later known as OPCs, as well as the late stage of OPCs, the OLs [28]. In the normal CNS of adults, most proliferating cells originate from the OPCs, which self‐renew through division and produce mature OLs, while there is little evidence to suggest that neurons can proliferate in adulthood [29]. Generally, the pluripotency of OPCs in the differentiation of other cell types, such as astrocytes, microglia, and even functional neurons, has been confirmed [30].

Indeed, OPC accounts for only 5–8% of total glial cells and is evenly distributed in WM and gray matter (GM), with slightly lower levels of OPC in GM. Position leads to behavioral differences in area regional OPCs [31]. In organotypic brain slices, WM NG2+OPC has a stronger proliferative response to platelet‐derived growth factor (PDGF)‐A, while GM OPC has a weaker response to PDGF‐A and is not as mature as WM OPC in morphology and genetics [32, 33]. Additionally, some research studies have reported that compared with GM OPC, WM OPC differentiates more into myelin OLs, many of which are still NG2+progenitor cells [34, 35], indicating the existence of a potential OPC reserve pool in adulthood (Figure 1B). In the adult CNS, the production of OLs by OPCs slows down. The OLs produced by WM OPCs account for about 20% of the total differentiated and myelinated OLs in the corpus callosum of mice, while they account for 5% in the cortex [36]. However, 20% of cortical GM OLs are differentiated as CNP+NG2 OLs, which do not form myelin sheaths. Recently, researchers have revealed that NG2+cells in cortex are highly dynamic, balancing their population through proliferation, differentiation, and self‐rejection [37]. Meanwhile, the common characteristic biomarker of OPC is identified as PDGFR‐α, the receptor for PDGF‐A, which is the OPC mitogen and survival factor, produced by astrocytes and neurons [38, 39] (Figure 1C). Therefore, overexpression of this growth factor, such as in developmental process, can lead to the increased OPCs numbers [40]. Additionally, the anterior OLs bind to their targeting axon, losing the bipolarity and beginning to establish filamentous myelin sheath growth. During the differential stage, the characteristic of OLs is the expression of three major myelin with different marker, such as 20,30‐cyclic nucleotide 30 phosphodiesterase (CNPase) and surface markers O4 and O1 [41]. Mature and differentiated OLs can be characterized by the production of myelin and myelin protein, combined with cell lineage‐specific markers (such as Olig2) to identify the mature stage [42]. In addition, myelin proteins include myelin basic protein (MBP) distributed on the cytoplasmic surface of the plasma membrane [43], transmembrane protein PLP [44], myelin‐associated glycoprotein [45], as well as galactocerebroside (GalC) [46] and also myelin OL glycoprotein (MOG) [47]. Moreover, PLP has recently been identified in mouse Olig2+PDGFR‐α+cells, making it an early marker of OPCs and acting a role in extension process [48]. The marker for the early OL lineage is proteoglycan NG2, while NG2+cells can differentiate into both OLs and astrocytes [49].

### 2.2 Neural Plasticity Regulation of OPCs and OLs

Actually, the neural plasticity regulation of OPCs and OLs due to their death in the CNS is related to several diseases, such as trauma, ischemia, or autoimmune attack commonly [50]. The death of OLs can result in subsequent demyelination and can also be a consequence of myelin previous injury [51]. In traumatic injury and ischemia, OL death and demyelination can occur with the original injury [52], while in autoimmune diseases, OLs are the main target of immune attacks against myelin and OL‐related proteins [53, 54]. Meanwhile, OPCs and OLs can interact with the neural microenvironment and exist in the form of synaptic connections. Additionally, OPCs and OLs can influence surrounding neural circuits, increasing neuronal excitability and activity. Neural signals from the microenvironment affect the expression of neuronal genes and cellular states, leading to heightened neuronal signaling and facilitating neuronal connections. Moreover, dynamic communication between neurons and glial cells (including OLs, OPCs, and astrocytes) is essential for the formation and maintenance of brain function during normal development and throughout the entire life cycle. Neural signals from the microenvironment affect the expression of neuronal genes and the cellular state of OL lineage cells. Here, the increase in neuronal signal transduction promotes neuronal connections between neural precursor cells (NPCs) and OL lineage cells (such as OPCs) and surrounded neurons.

It is already known that WM pathology is a feature of AD, while OLs death and demyelination are thought to be secondary to neurodegeneration [55]. Either the decreased number of OPCs or myelin OLs were found in AD [56]. Although the cause has not been determined, the toxicity of β‐amyloid protein may be a main factor [57]. However, research has suggested that OPC and OL pathology can be proven even before another neurodegenerative event occurs [58]. It is interesting that the WM pathology of AD mainly affects the CNS region where myelin sheaths are formed during development, indicating a link between late myelin development and AD [59]. Although not considered to be the primary cause, OL pathology is a downstream target for a number of neuropsychiatric disorders, including schizophrenia, bipolar disorder (BD), autism, attention deficit hyperactivity disorder (ADHD), mood disorders, and depression [60, 61]. For example, there is a region‐specific decrease in OL density in patients with BD and schizophrenia [62, 63]. Research on cuprizone model, showing as the demyelinating model, has also been used to simulate various aspects of schizophrenia and anxiety disorders [64]. Additionally, OL‐specific genes in the amygdala and prefrontal cortex are downregulated in chronic stress model [65], while dysregulation of OLs in Ranvier lymph nodes is associated with depression [66].

**FIGURE 2:**
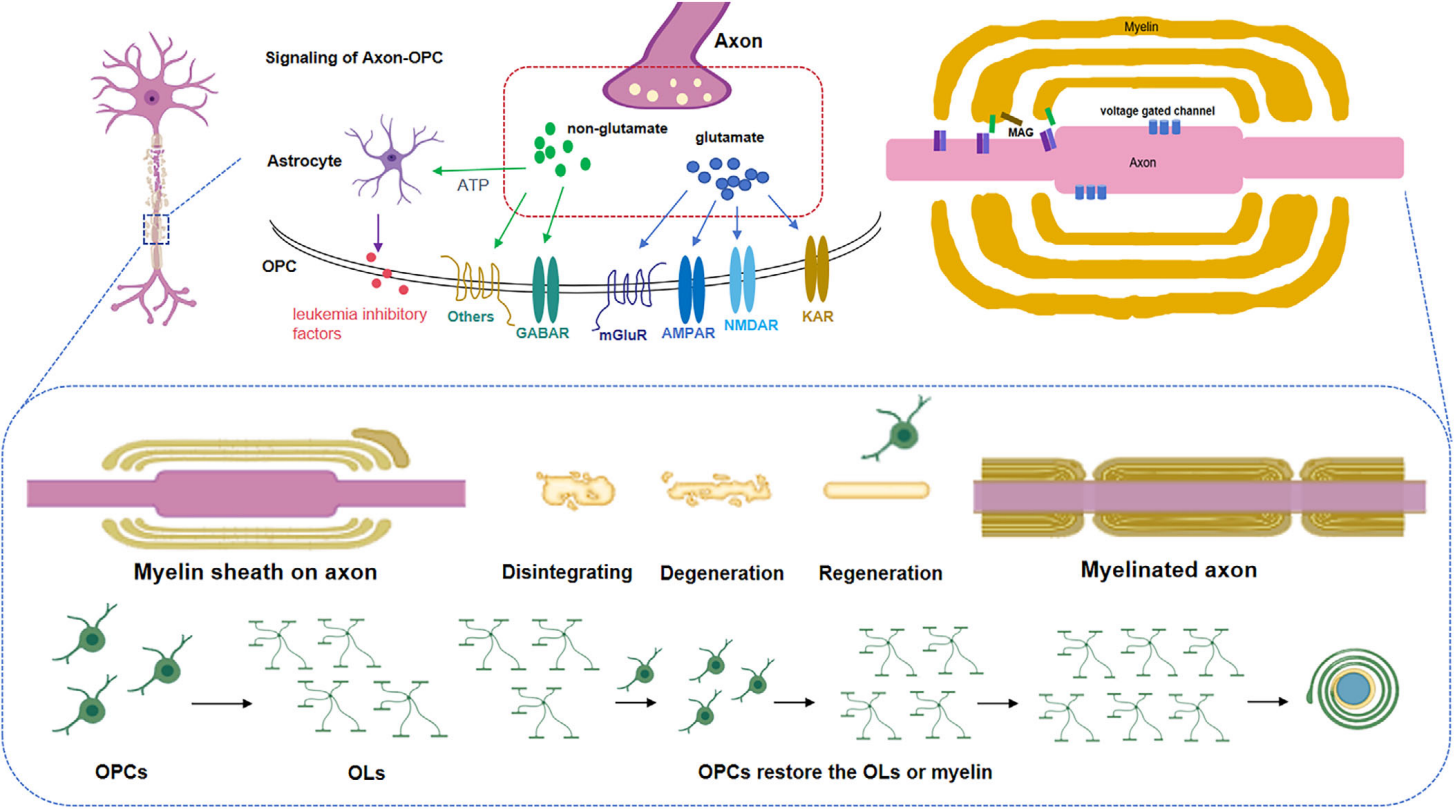
The process of myelination, demyelination, and remyelination. OLs myelinate large diameter axons in the CNS and provide trophic support for the underlying axon. OLs are absent and can lead to the demyelination (death of OLs) in certain pathological states, such as injury, trauma, or ischemia. New OLs derived from OPCs can replace deceased OLs, which can reinstate the myelin sheath around demyelinated axons (remyelination). Neurotransmitters including glutamates and GABA or others such as ATP from WM and GM axons can interact with synaptic (AMPAR or NMDAR) or other‐synaptic (mGluR or KAR) receptors in OPCs. And also, ATP can act on OPCs by astrocytes, releasing factors such as leukemia inhibitory factors (LIF) for stimulating the synthesis of myelin. During postnatal development, these signals stimulate the production of myelin by enhancing the survival of newly formed myelin.

Actually, MS is also the demyelinating disease and characterized by immune‐mediated attacks on myelin and OLs, mainly myelin‐specific CD8+ T cells [67]. This leads to demyelination, characterized by myelin sheath destruction and death of Ols [68], causing axonal detachment and susceptibility to neurodegeneration [69]. The mature OPCs are the major source of new OLs after myelin injury and make up about 6% of the total number of cells in the CNS. In the demyelination process, OPCs are activated and characterized by increased transcription factors (TFs) expression [70], promoting proliferation and colonization of demyelinating regions [71]. OPCs move to the lesion site under the guidance of either microglia or astrocyte derived factors (such as brain‐derived neurotrophic factor [BDNF] and FGF) [72], and then exits the cell cycle under the guidance of TFs, differentiating into mature OLs [73]. These new OLs can replace damaged myelin with shorter and thinner ones. While, due to the decrease in recruitment and differentiation of OLs by OPCs with aging, which subsequently fail to differentiate into OLs [74]. Therefore, dead OLs are not replaced efficiently in old age, leading to the WM atrophy (Figure 2).

### 2.3 The Biological Function Related to OL Lineage

Generally, under healthy conditions, multiple interactions between neurons and glial cells including OPCs and OLs promote synaptic formation and cell proliferation. Similarly, in disease states, the interplay between neurons and OPCs and OLs in the relevant microenvironment serves as a critical mechanism for the development of OPCs and OLs. In addition, the cellular state is also crucial for NPC/OPC cells to receive signals from the microenvironment. Microglia are the innate immune cells of the CNS. Their activation represents the early events of disease development, loss of BBB function, and inflammation. It is worth noting that under pathological conditions, the balance between proinflammatory and anti‐inflammatory factors in the body is disrupted, leading to persistently high levels of proinflammatory factors and inducing chronic inflammation. This chronic inflammatory state is also the common soil for related neurodegenerative diseases. All of those multiple factors has a negative impact on OLs and myelin sheaths.

Surprisingly, there are evidence that suggest that OPC also has immunomodulatory abilities [75]. OPC expresses cytokines receptors [76] were evaluated for their microenvironment through filamentous foot extension [77]. Meanwhile, corresponding to inflammatory signals, OPCs can migrate to the injury sites similarly as microglia [78]. When IFNγ stimulation, OPC presents antigens to cytotoxic CD8+T cells, resulting in cytotoxic cell death [79]. And these proinflammatory OPCs accelerate tissue damage and block myelin regeneration, indicating that inhibited OPC‐mediating inflammatory process can restore cell death and promote OPCs differentiation into OLs [80]. The OPCs and OLs isolation from mouse CNS tissues are based on the mature stage of expression of such typical markers as PDGF‐α or A2B5 for OPCs; O4 or GalC for OLs [81, 82]. Obviously, the primary culture is used for studying OPC proliferation, survival, and differentiation, as well as the impact of interested molecules on the biology of OPCs and OLs. In addition, there are different methods for studying myelin formation by coculturing OLs with neurons or synthesizing nanofibers [83]. Meanwhile, the primary OPC culture is also suitable for high‐throughput screening of pharmacological compounds that may interfere with OPC [84]. However, the cultivation of primary OPC is subject to issues of animal availability and ethical constraints. Due to these limits, several research groups have developed spontaneously immortalized cell lines, such as CG‐4 or OLN‐93 immortalized OPC [85, 86]. The immortalized cell line has undeniable advantages and low cost, but due to the unpredictability of mutations that lead to immortalized cells, any hypothesis and translation of physiological function need to be confirmed through additional models. The possibility of using OPCs obtained from patients with neurological diseases as research models has made pluripotent stem cells (iPSC) popular [87], as evidenced by the various protocols developed. In addition, the possibility of OPC differentiation toward iOL is suitable to study this process applied in human system [88]. iPSC are reprogrammed from somatic cells and can subsequently be differentiated into any cell type, either by specific growth factors (e.g., PDGF‐A to OPCs) or through inducing TFs expression, which is required for transformation [89, 90, 91]. On the other side, simulation of the OPCs and OLs located environment such as mixed glial cell culture [92], organoid brain slices [93], and iPSC‐derived organoids [94], which could help to investigate the effects of other cell types on OPCs and OLs in vivo studies. To date, OPCs have been shown excellent potent in clinic due to an increased recovery rate, decreased side effects, and the achievement in the tissue injury. While OPCs, in addition to their myelination attribute, have been recognized as important factors in regulating neuronal function in the CNS (Figure 2).

### 2.4 Therapeutic Applications of OL Lineage and Their Targets in Diseases

#### 2.4.1 Strategies of Delivery to CNS System

**FIGURE 3:**
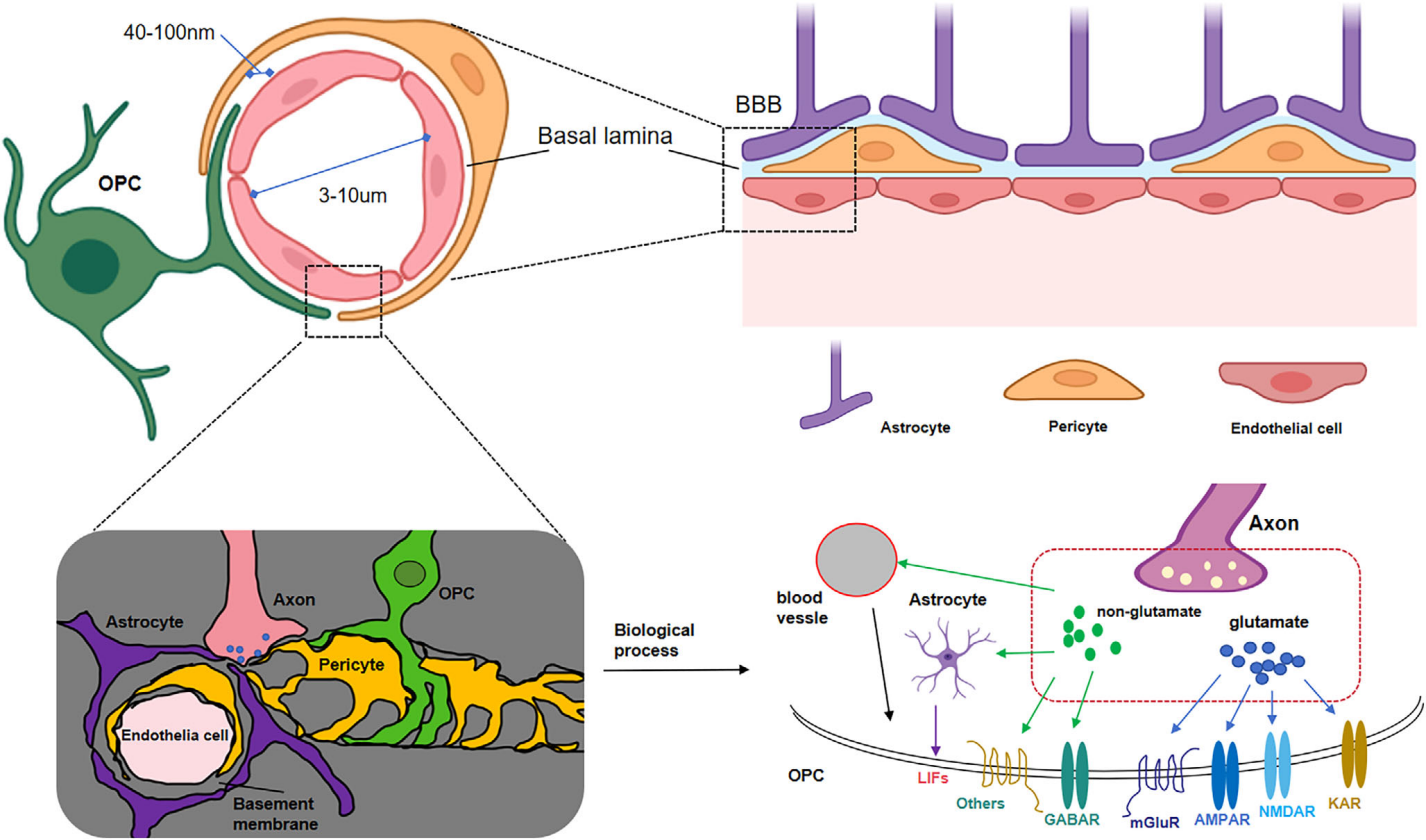
The properties of the neurovascular unit includes an intact BBB that displays multiple characteristics that limit drug permeability into the CNS. The blood–brain barrier (BBB) structure displays exhibits physical properties regulating molecular and cellular flow in the neural parenchyma. In addition, Axons can also stimulate blood vessels to release endothelin, thereby affecting the synthesis of individual OLs.

As mentioned earlier, in CNS, neuropsychiatric diseases are the greatest challenges in the world [95].In addition, the neurological diseases including AD and PD, which greatly affect the elder population in China [96]. Meanwhile, biomaterial‐based delivery systems represent a potential pathway to enable new treatments for CNS diseases [97]. Particularly for patients with brain tumors or cancers with metastases to brain, they have few options rather than surgical operation, chemotherapy, and radiation [98]. To date, drug delivery strategies to CNS can be achieved through intravenous administration, invasive local administration (including intrathecal injection, intracerebral parenchymal administration), and intranasal as well as peripheral administration [99]. Intravenous injection provides the minimum extent possible invasive chance for drugs to enter the brain, but this requires crossing the BBB. As shown in Figure 3, the presence of BBB prevents large molecules, except for small ones, from entering the brain through systematic drug delivery [100]. At present, it is possible to transendocytosis of cerebral endothelium through synthetic agents [101]. Biocarrier delivery and temporary disruption of the BBB to enhance drug delivery from the blood circulation to the brain [102].

#### 2.4.2 Endothelial Cells Targeted Delivery

**FIGURE 4:**
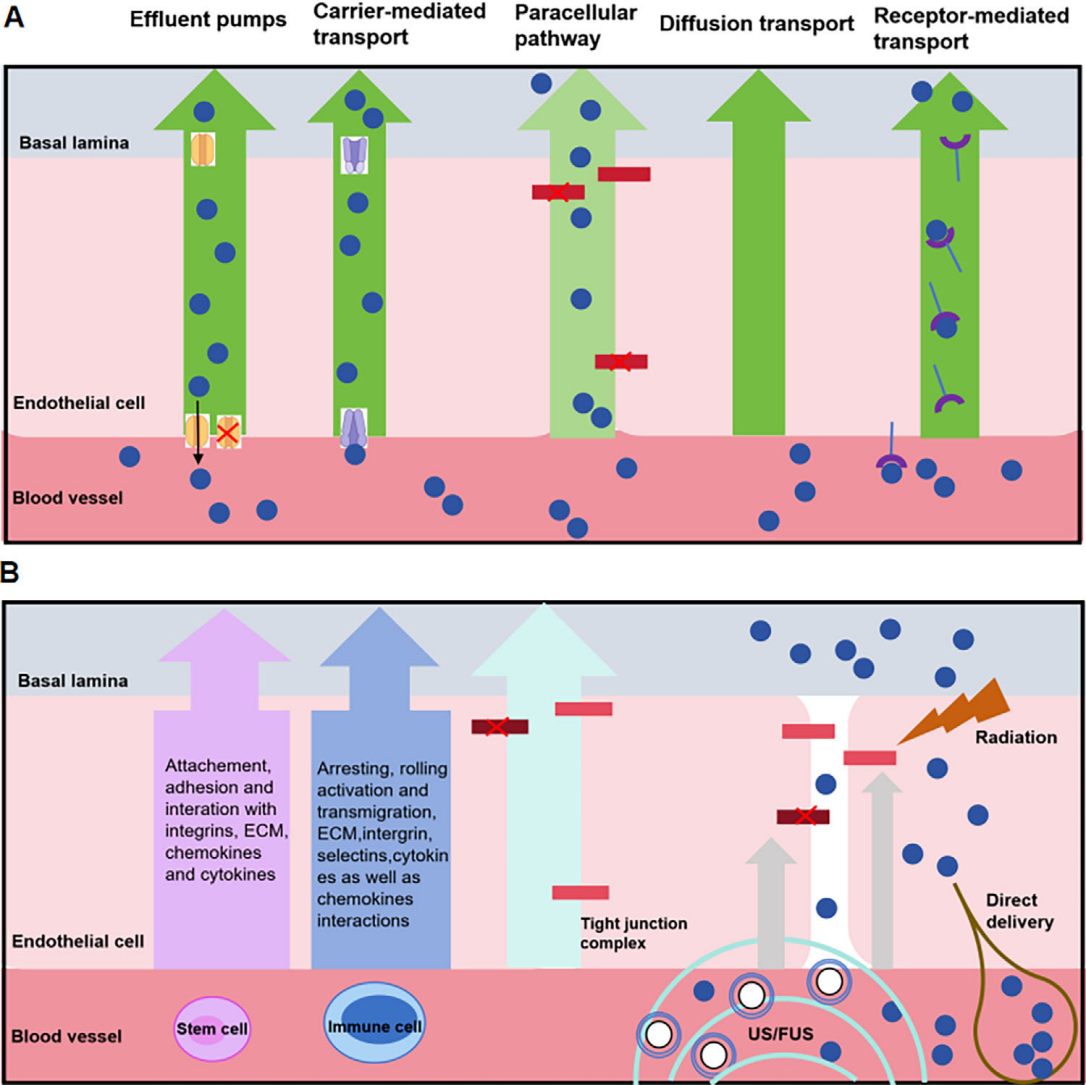
The multiple strategies/pathways are employed to overcome BBB. (A) The molecular strategies/pathways are applied to overcome BBB. The drugs or therapeutics agents (blue circles) can be designed with either affinity to efflux pumps or receptor/carrier‐mediated transport mechanisms as well as paracellular pathways by junctional complexes. (B) The cellular and direct delivery strategies/pathways to bypass BBB. The immune cells could transmigrate to the CNS mediated by actin‐containing protrusive structures. And also stem cells that have been modified and displayed transcellular or paracellular transmigration. Directly, ultrasound or focused ultrasound (US/FUS) with microbubbles (concentric small blue circles), radiation could mediate direct delivery of therapeutics to the CNS. Reproduced with the authors permission [186]. Copyright 2020. HHS Public Access.

As discussed earlier, brain endothelial cells formed the neurovascular unit that regulates the substances transport by transporters, receptors, and efflux pumps across the BBB (Figure 4A and Table 1). It is worth mentioning that in some disease states, the expression of transporters and receptors may be altered, thus affecting drug delivery vectors using these pathways [103]. For example, in AD, the expression of low‐density lipoprotein receptor‐associated proteins is reduced, while the upregulated expression of some drug efflux transporters is observed [104].

**TABLE 1:** Cellular and molecular approaches to across the BBB.

Approach	Strategy/pathway	Obtained and comments	References
Cellular	Paracellular and transcellular	The transporters, receptors, and efflux pumps, which could be used for drug delivery across the BBB	103, 104
Receptor mediated	Transcellular (transcytosis and diffusion)	Ligand–receptor interaction mediated drug delivery targeting the specific cell type in CNS	155, 156, 157, 158, 159, 160, 161
Channel mediated	Trans cellular	Channels were a potential, promising target and could increase drug delivery selectively to brain	205
Gap–junctional complex	Tight junction pathways	Downregulation of tight junction protein expression in BBB leaded to space dilation around blood vessels	206

#### 2.4.3 Brain‐Vasculature Targeted Delivery

Although the development of BBB endocytosis transporters has achieved great success, its overall delivery efficiency is very low, and it is mostly related to the microvascular endothelium in the brain [105]. For example, after intravenous administration, more than 90% of OX26 was found in brain capillaries rather than in the retrocapillary chamber [106]. In addition, functionalized liposomes loaded OX26 that bind to brain capillaries without endocytosis could deliver oxaliplatin encapsulated to brain [107]. Therefore, the cerebrovascular system can also be considered as a storage place for drug delivery to the brain. At the same time, red blood cells, extracellular vesicles, exosomes, and so on can be used as carriers to deliver drugs to the brain [108, 109, 110]. In addition, drug delivery can be achieved by using nondestructive pathways to regulate the BBB [111] or by temporarily disrupting the BBB [112]. For example, magnetic resonance is used to guide and optimize perfusion areas affected by penetrant delivery [113], combined with short interruptions to reduce adverse side effects. Focused ultrasound, regulated by precise time and space, can temporarily breach the BBB [114]. In combination with MRI, MRgFUS improves the brain's delivery of small molecule, proteins, and synthetic nanoparticles, viruses as well [115]. For instance, microbubble (MB)‐loaded drugs are mediated by ultrasound [116], and microvesicles temporarily increase neurovascular permeability by responding to ultrasound [117]. Additionally, An et al. showed a one‐stone‐two‐birds strategy of targeting MBs with “dual” anti‐inflammatory and BBB “switch” function combined with ultrasound for ischemic stroke treatment [118]. Therefore, either ultrasound or focused ultrasound is a promised method for targeted delivery to brain (Figure 4B and Table 2).

**TABLE 2:** Invasive approaches to across the BBB.

Approach	Strategy/pathway	Obtained and comments	References
Ultrasound with microbubbles	Paracellular and transcellular (diffusion and convection)	Revealed microbubble/microvesicles loading mediated by ultrasound is a promising method for targeted delivery to specific locations within the brain.	117, 118
Nanoparticles	Paracellular and transcellular (diffusion and transcytosis) Also combined with radiation	Demonstrated the enhanced delivery efficiency and blocked the active output of BBB receptors and prevented degradation	123, 124, 125, 126, 127, 140, 141, 142, 143

#### 2.4.4 Intranasal and Peripheral Delivery

Compared with the above two routes of administration, intranasal administration can be delivered to the brain bypassing the BBB [119]. For example, some lipophilic drugs and biologics may leak into the brain and cerebrospinal fluid through the nasal epithelium, or transneuronal pathways along the olfactory and trigeminal axons [120]. Compared with systemic or local administration, intranasal administration has the advantage of being easy to use, with faster drug onset and greater bioavailability, reducing systemic exposure [121]. However, the disadvantage of this method is that it is limited by the surface area of the nasal cavity and the characteristics of the nasal mucosa, which reduces the effective absorption of the drug [122]. The use of surfactant or nanoparticle packaging to enhance delivery is currently being investigated. For example, either alginate or chitosan nanoparticles can block the activated output of BBB receptors and prevent degradation [123]. Nanostructured lipids, nanoemulsions, and chitosan coating can be used to improve intranasal delivery to the brain [124]. Biodegradable polymer materials such as polylactic acid, polyglycolic acid, PLGA, and polysebacic anhydride encapsulate drugs to enhance their stability for intranasal administration [125, 126, 127]. Targeted uptake and delivery to the nasal epithelium can be further achieved through the functionalization of lectins, cell‐penetrating peptides, and proteins [128].

Similarly, peripheral injections can also be ingested by the motor neurons and autonomic nervous system and delivered to the central nervous system and spinal cord [129, 130]. Intramuscular injection can also be delivered to the CNS through retrograde transport of nerve axons [131]. Delivery of viral and nonviral biological material to the CNS by intramuscular injection and retrograded‐transporting has now been investigated in animal models [132].

#### 2.4.5 Invasive Local Delivery

In surgical interventions for the resection of malignant tumors, subarachnoid hemorrhage, PD, or the treatment of traumatic injuries, local or nonsystemic routes of administration are often invasive [133]. For example, hydrogel scaffolds, polymer films, microspheres, or nanoparticles can be implanted directly into the brain parenchyma [134, 135, 136].

Up to date, natural or synthetic polymers can be injected into the brain parenchyma for drug delivery, and these systems provide real‐time controlled and long‐lasting delivery as the component material degrades [137]. As mentioned above, natural polymers including alginate, hyaluronic acid, and chitosan, etc. and proteins such as collagen, albumin, gelatin, etc.[138], which have been used in wafers, hydrogels, hydrogel scaffolds, conductive polymers and particles as well as nanoparticles [139]. On the other hand, biomaterials injected directly into cerebrospinal fluid can bypass the BBB and have less off‐target and toxicity with high dose administration, so intravaginal delivery can avoid the deficiencies of systemic drug delivery and nonviral gene delivery. For example, nanoparticle and polymer formulations combined with radiation are being explored to improve delivery efficiency and penetration outside brain tissue [140]. Additionally, polyvinylimine–DNA complexes, liposomes, and nanoparticles have been used for in vivo delivery of siRNA and nonviral genes [141]. At the same time, more multifunctional polymer materials are optimized to improve the stability of drug delivery and promote endosome escape [142]. In addition, the copolymer was engineered to form like a virus, increasing the ability to deliver genes to the brain [143] (Table 2).

#### 2.4.6 Cell Membrane‐Coated Delivery

The cell membrane‐coated drug delivery system that has been developed fast in recent years and several researchers have reported this new technologies including the synthesis and application in the related fields [144, 145, 146]. Among those, the nanoparticles coated with different cell membranes exhibit some different properties. For example, the RBC membrane coating, exhibiting the prolong time in circulation [147]; the platelet membrane coating showing the strong ability to adhesion to the vessel wall after injury and interact to the extracellular matrix (ECM) [148]; the tumor cell membrane coating displaying the specific immune response [149]; similarly, the bacterial cell membrane coating showing the antibacterial immunity [150]. In addition, the MSC membrane coating also shows nice tumorigenicity [151]. Considering the various types of proteins including the useful ligands and receptors expressed on the MSC membrane [152], the MSC membrane is receiving more attention and also the stem cell therapy based on MSC has already been used as common treatment in many countries [153]. Therefore, modifying MSC membranes will be a fundamental strategy for manufacturing membrane‐coated delivery system/platform with specific functions compared with other cell membranes [154] (Table 3).

**TABLE 3:** The proteins and receptors in cell membrane for crossing BBB.

Type	Strategy/pathway	Proteins and receptors	References
Stem cell	Adhesion and interaction	Integrinɑ Growth factors: TGF‐b, HGF ECM‐receptors: CD45, CD47 Cytokine: TNF, IFN Chemokin: CXCR1, CXCR4, CCR1	148, 156
Immune cell	Arresting, activation, and transmigration, interaction as well	P‐selectin, E‐selectin Integrinɑ ICAM‐1, VCAM1 Cytokine: TNF, IFN Chemokin: CXCR1, CXCR4, CCR1	157, 158, 159, 163

Materials glycosylphosphatidylinositol as the widely used biomaterials can be attached to the cell membrane through the lipids interactions [155], such as using liposomes fused with cell membrane, which facilitate the inclusion of probes, proteins/receptors as well as some drugs within the membrane. Moreover, these liposome‐coated membranes with transmembrane proteins, for instance, cytokines (CD45, CD47) [156], P‐selectin glycoprotein ligand‐1 [157], lymphocyte function‐associated antigen‐1 [158], as so on, show the strong chemotaxis ability to overcome the vascular barrier and target to the inflammatory site [159]. On the other side, cell membranes can receive different covalent conjugation reactions and several functional groups can be directly conjugated to the membrane surface, such as molecules, ligands, and nucleic acids with those groups [160]. PEG has been applied as the conjugation linker, which provides enough space for the ligand–receptor interaction and has a critical role in uptake of cells [161]. Parallelly, MSC membranes have been modified that present very good immuno‐suppressive effects and homing ability [162]. Therefore, these modified MSCs display the potential of immunomodulatory agents delivery [163].

**FIGURE 5:**
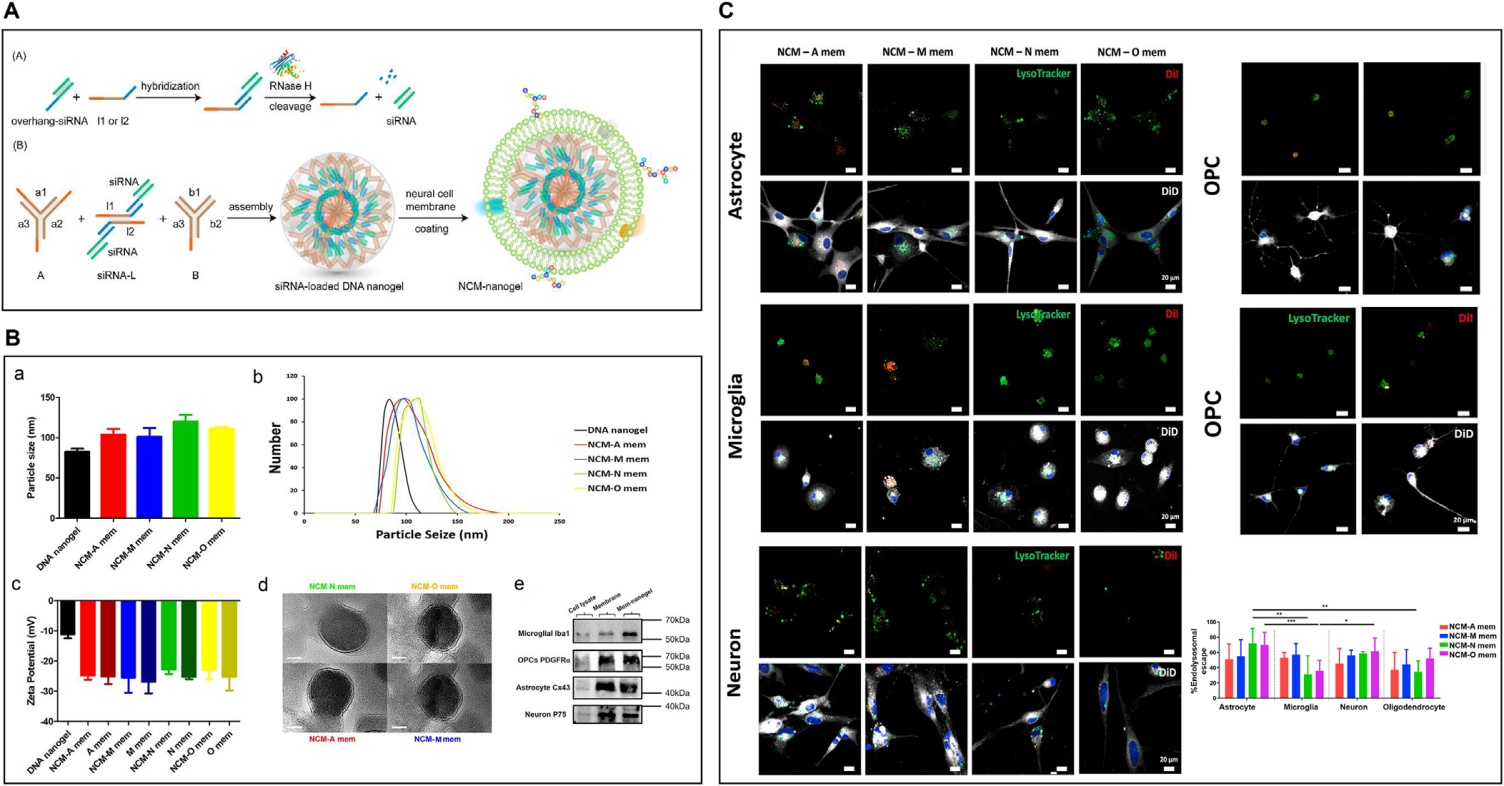
Neural cell membrane‐coated DNA nanogels as a potential target‐specific drug delivery tool for the central nervous system. (A) Schematic representation of siRNA hybridized with l1 or l2. (B) Schematic illustration of self‐assembly of building blocks A, B, and siRNA‐L into DNA nanogel, and its subsequent NCM coating. (B) Successful neural cell membrane coating over DNA nanogels as shown by increased size of DNA nanogels, increased negative surface charge and presence of core–shell structures under the TEM. (C) NCM‐N mem and NCM‐O mem showed higher endolysosomal escape in astrocytes. and quantitative data of endolysosomal escape of neural cell membrane‐coated DNA nanogels within astrocytes, microglia, neurons, and oligodendrocytes (*n* = 3). *: *p* < 0.05; **: *p* < 0.01; ***: *p* < 0.005. One‐way ANOVA. At least 10 ROIs including more than 100 cells were quantified for each repeat. Reproduced with permission [166], 2023, Elsevier Ltd.

Similarly, synthesis of micro/nanoparticles coated by biological cell membranes has become a new type of drug delivery carrier [164] (Table 4). Drug delivery targeting the specific cell type in the CNS plays a critical role in the treatment of neurological diseases [165]. Zhang et al. have reported that neural cell membrane‐coated nanoparticles could target and enhance the ability of uptake by CNS cells [166]. Meanwhile, Lin et al. observed a selective uptake of OPC membrane‐coated DNA nanogels (NCM‐O mem) by OLs both in vitro and in vivo studies. In addition, the selective and effective gene knockdown capacity of NCM‐O mem for OPC transfection was confirmed by qPCR [167] (Figure 5). Additionally, Lampe group investigated the delivery of PDGF‐AA, from poly(lactic‐co‐glycolic) acid microparticles to OPCs in a 3D polyethylene glycol‐based hydrogel, which is quite similar to the native brain environment and detected the relationship between the encapsulated OPCs fate and PDGF‐AA release kinetics [168].

**TABLE 4:** Characteristics of types of vesicles for delivery to brain.

Type	Size	Advantages and limitations	References
Membrane	100–150 nm	Bypass the BBB, high yield	11, 14, 16
Nanovesicles	100–150 nm	Bypass the BBB, long time in circulation, immune escape and high yield; but only with membrane protein functions	22, 23, 24
Microvesicles	100–1000 nm	Bypass the BBB but complicated extraction and high cost	103, 104
Exosomes	40–160 nm	Bypass the BBB, long time in circulation, immune escape; but complicated extraction and high cost	155, 156, 157, 158, 159, 160, 161

## 3 The Therapeutic Role of OL Lineage in CNS Diseases

### 3.1 OL Lineage Applied in Nerve Injury

As is discussed earlier, OPCs as NSCs have inherent tumorigenic and inflammatory migratory properties; those characteristics provide significant advantages over other cells [169]. OPCs have been used in tissue regeneration, antitumor therapy based on these properties [170]. Similarly, anti‐inflammatory research has shown that nanoparticles loading with OPCs and OLs also exhibit targeting functions for inflammatory sites [171].

**FIGURE 6:**
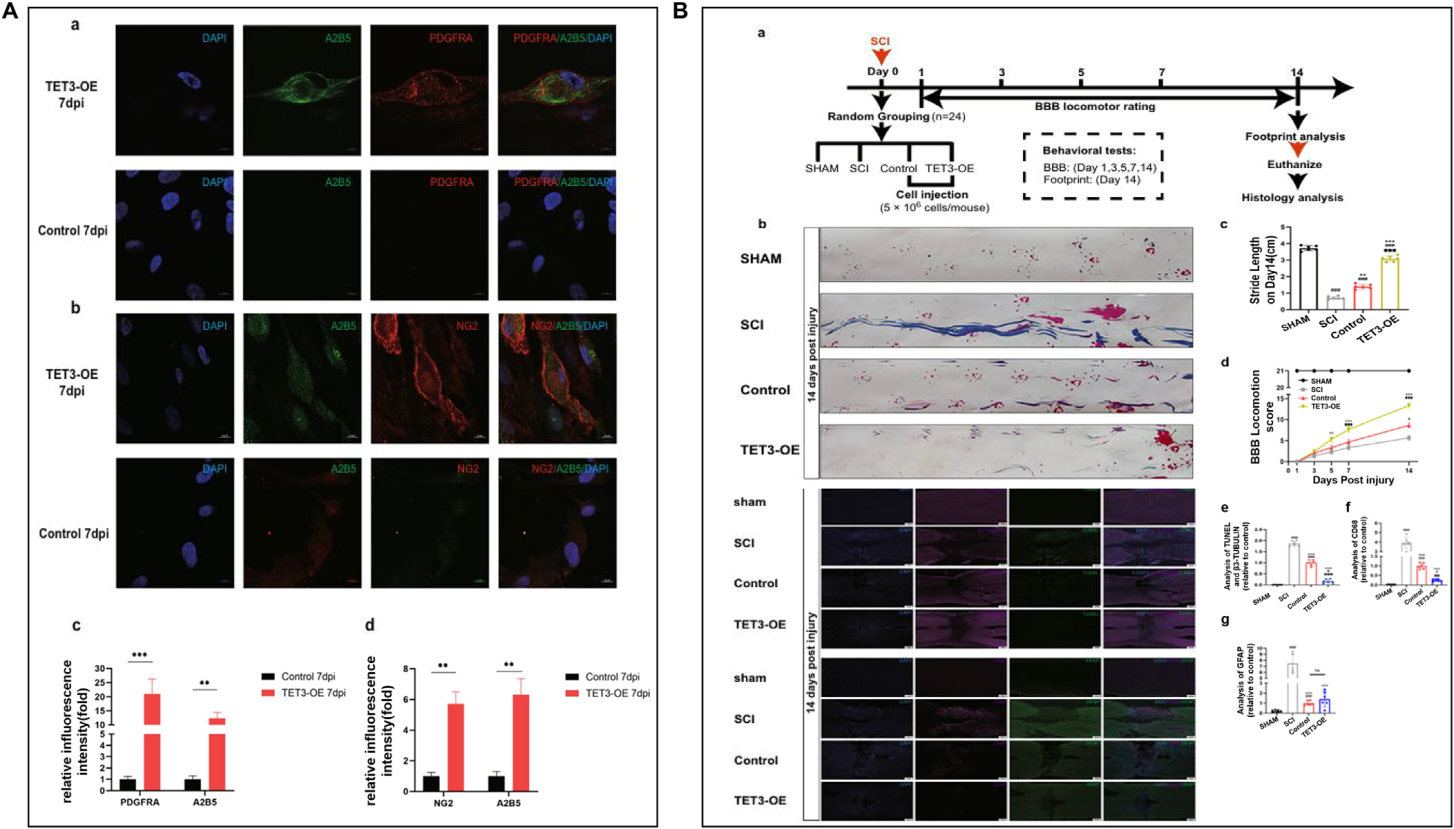
Investigation of the ability of TET3 to induce HUCMSCs into OPCs and to promote recovery of SCI rats treated with TET3‐OE [181]. (A) Confocal imaging of iOPCs. (a and b) At 7 dpi, the expression patterns of NG2, PDGFRA, and A2B5 in TET3‐OE display localization resembling that of OPCs. (c and d) The expression levels of NG2, PDGFRA, and A2B5 in TET3‐OE are significantly higher than those in control (*n* = 6 samples, scale bar = 10 µm). Data were shown as mean ± standard deviation. Statistical analysis was performed unpaired *t*‐test, **p <  0.01, ***p < 0.001. (B) SCI model rat recovery improved when treated with TET3‐OE and double immunostaining of TUNEL and β3‐TUBULIN; GFAP and CD68 at 14 days postinjury (scale bar = 500 µm, *n* = 6 rats). (a) Experimental timeline for the rat spinal cord injury (SCI) model. Eight‐week‐old female Sprague–Dawley rats (*n* = 24; 250–300 g) were randomly divided into four groups: SCI, sham, control, and TET3‐OE. SCI was induced using Allen's method on Day 0. Intrathecal injections (5 × 106 cells) were administered immediately after surgery. Behavioral assessments using the Basso, Beattie, and Bresnahan (BBB) locomotor rating scale were performed on Days 1, 3, 5, 7, and 14 postsurgery. On Day 14, footprint analysis was conducted, followed by euthanasia. Spinal cord tissues were collected for histology analysis. (b and c) Footprint analysis on Day 14 postinjury showed that the TET3‐OE group exhibited the most significant improvement (*n* = 6 rats). Representative images for each group were randomly chosen, avoiding selection of the most severe or mild cases. Data were shown as mean ± standard deviation, statistical analysis was performed using one‐way ANOVA with Tukey's HSD posthoc test. **p < 0.01, ***p < 0.001 versus SCI group, ###*p* < 0.001 versus sham group, ■■■*p* < 0.001 versus TET3‐OE group. (d) The Basso, Beattie, and Bresnahan score showed significant locomotion function recovery in the TET3‐OE group (*n* = 6 rats). Data were shown as mean ± standard deviation. Statistical analysis was performed using two‐way ANOVA with repeated measures, followed by Sidak's test. *p < 0.05, **p < 0.01, ***p < 0.001 versus SCI group, ###*p* < 0.001 versus sham group, ■■■*p* < 0.001 versus control group. (e) Quantitative analysis of TUNEL and β3‐TUBULIN double staining cells showed reduced neuronal apoptosis in the TET3‐OE group. (f) Quantitative analysis of CD68 showed fewest macrophages infiltration in the TET3‐OE group. (g) Quantitative analysis of GFAP demonstrates reduced astrocyte reactivity in the TET3‐OE group and control group. Data were presented as mean ± standard deviation. Statistical analysis was performed using one‐way ANOVA with Tukey's HSD posthoc test. *p < 0.05, **p < 0.01, ***p < 0.001 versus SCI group, #*p* < 0.05, ###*p* < 0.001 versus sham group, ■■*p* < 0.01, ■■■*p* < 0.001 versus TET3‐OE group. ns, *p* > 0.05 between TET3‐OE group and control group. Reproduced with permission [181]. The Author(s) 2024. Open Access.

Traumatic brain injury and spinal cord injury (SCI), two types of neurotrauma, are one of the most challenging clinical problems due to their complex pathophysiological factors and their development over time, which increases the difficulty of finding appropriate treatments [172, 173]. Besides the primary trauma, secondary injuries lead to further damage, but these also provide many potential avenues for intervention [174]. Inflammation is an important part of the secondary injury, resulting in further damage and cells death [175]. In animal models, reducing posttraumatic neuro‐inflammation promotes improved neurological function [176]. Even though anti‐inflammatory drugs may be suitable in nerve trauma treatment, there is still a need to optimize the administration method to reduce systemic side effects [177]. For instance, steroids as anti‐inflammatory agents can be addressed both locally and by achieving locally controlled drug release through polymer‐based approaches [178]. In addition to the acute phase, the chronic phase of SCI provides chance to enhance recovery through rehabilitation and neuromodulation [179]. Increased tissue retention after injury can greatly enhance the effects of some emerging rehabilitation treatments, which can be further improved. For example, Raffaele et al. have shown that microglia play a beneficial role in promoting OPC recruitment to ischemic lesions and maintaining myelin integrity and that microglia communicating with OPCs act as the promising effect on myelin regeneration and functional recovery after stroke [180]. Additionally, Zhang et al. have suggested that the demethylation mediated by tet3 reshapes the methylation pattern of HUCMSCs, enabling efficient one‐step conversion to OPCs and significantly reducing the time required for cell preparation after SCI. And also, their protocol could help to promote cell therapy strategies for SCI from the perspective of methylation regulation [181] (Figure 6).

In neurotrauma, OPCs proliferate rapidly, but most fail to differentiate into mature OLs in time, resulting in insufficient myelin regeneration. This is similar to the pathological features in demyelinating diseases such as MS, suggesting that the obstruction of OPC differentiation is a key factor for the limited functional recovery after nerve injury. In addition, the differentiation of OPCs into mature OLs and the formation of myelin sheaths are necessary conditions for the efficient transmission of neural signals. If the OPC cannot differentiate effectively after nerve injury, it will lead to a slowdown in axonal conduction velocity and affect the recovery of nerve function. Therefore, promoting OPC differentiation and myelin regeneration is the key to improving function after nerve injury. In fact, although a large number of brain and spinal cord organoids have been constructed and applied in nervous systems, there remains a lack of OL lineage‐specific organoids/tissue‐like constructs suitable for repairing neurological injuries. The reason is that the main function of the OL lineage is considered to be myelin formation, rather than directly participating in axonal projection processes. Research in bench provides a theoretical basis for the development of therapeutic strategies for nerve injury. For instance, by regulating the specific signal, controlling the function of microglia, or promoting the differentiation of OPCs, it is expected to enhance the repair effect after nerve injury, especially in the treatment of CNS injuries such as nerve injury and SCI, which has potential application value.

Up to now, iPSCs have been reported, which can promote axonal regeneration and repair peripheral nerve injuries. This iPSC‐derived organoid was obtained by preparing motor and sensory axonal bundles, isolating organoid spheroids, and placing the axonal bundles into collagen gel conduits [182]. This type of peripheral nerve organoid contains straight axonal bundles, but the ECM composition and structure of peripheral nerves are not fully simulated, and the most critical myelin‐forming cells are absent. Additionally, the fractured and disintegrated axonal bundles posttransplantation can activate the host's immune system. Therefore, there is an urgent need to further apply tissue engineering principles to develop highly biomimetic neural 3D organoid. This would help mitigate negative effects affecting neural regeneration and facilitate research into neural defect repair mechanisms and the development of novel therapies. Again, the behavior and differentiation status of OL lineage after nerve injury directly affect the recovery of nerve function. A deep understanding of the regulatory mechanism of OL lineage is crucial for the development of effective nerve repair therapies.

### 3.2 In Antitumor Studies

Glioma is one of the most common types of primary brain cancer, among which GBM is the most aggressive and common, with a median survival time of only about 1 year [183]. Despite the protection of the BBB, about 9–17% of cancers metastasize to the brain. At the same time, because of the existence of the BBB, the delivery of drugs to the tumor site is limited, increasing the difficulty of brain cancer treatment [184]. Meanwhile, changes with brain cancer, including vascularization [185], ECM [186], and local immune composition, both impede drug delivery and can be used for drug delivery [187].

In general, there are dysfunctional blood vessels in GBM, containing more irregular blood vessels and increased permeability [188]. Increased permeability is related to the degree of tumor metastasis, but it also allows drugs to enter the brain tumor from the blood circulation. The researchers have proved that MRgFUS combined with MBs could activate the homing and differentiation of monocytes, resulting in the immune environment shift to the proinflammatory state in GBM [189]. In addition, glioma cells reshape the local ECM and produce some abnormal proteins in brain. Accordingly, materials that are reactive to remodeled ECM or upregulated proteases have been performed for tumor‐targeted drug delivery [190, 191]. At the same time, some factors secreted by glioma cells, which recruit immune cells into brain tumors, Thus, the migration of those immune cells from the blood circulation is also used for drug delivery [192].

As mentioned earlier, it is generally accepted that OPCs or NPCs may be the origin cells of GBM. Moreover, different cellular states lead to different evolutionary trajectories of GBM, which are influenced by the genetic variations of tumor cells themselves as well as the differences in the tumor microenvironment. Actually, in a healthy brain, the bidirectional interaction between neurons and glial cells promotes synaptic formation and glial cell proliferation. Similarly, in glioma, the interaction between neurons and glioma cells in the tumor microenvironment is also an important mechanism for the occurrence and development of GBM. Gliomas can interact with tumor microenvironment, which promote growth and resist therapy. Gliomas in form of the synaptic connections and neuronal activity increases tumor cells growth and invasion. Gliomas can also affect surrounding neural circuits, increasing neuronal excitability and activity. Neural signals from the tumor microenvironment affect the expression of tumor neuronal genes and cellular states. Herein, increased neuronal signaling promotes neuronal connections between NPCs and OL lineage cells such as OPC and surrounding neurons in tumor microenvironment [193, 194]. In addition, the dynamic communication between neurons and glial cells, including OLs, OPCs, and astrocytes, is crucial for the formation and maintenance of brain function during normal development and throughout the life cycle. Meanwhile, cell state is also crucial for NPC/OPC cells to receive signals from the microenvironment. To be noted, the research has reported that GBM patients with high neural signatures exhibit reduced overall and progression‐free survival compared with those with low neural signatures [195].

Indeed, gliomas exploit multiple neuronal interaction mechanisms to coproduce the tumor growth, invasion, and therapy‐resistant environment. However, the mechanisms of neuronal/GBM interactions, as well as therapeutic strategies, require further explored. With the increasingly refined research on the formation mechanism of the structural and functional network of neurons and glioma cells, the interaction between neurons and GBM cells and the mechanism by which tumor cells hijack neurons to promote tumor development are potential directions for GBM treatment.

### 3.3 In Neurodegenerative Disease Studies

**FIGURE 7:**
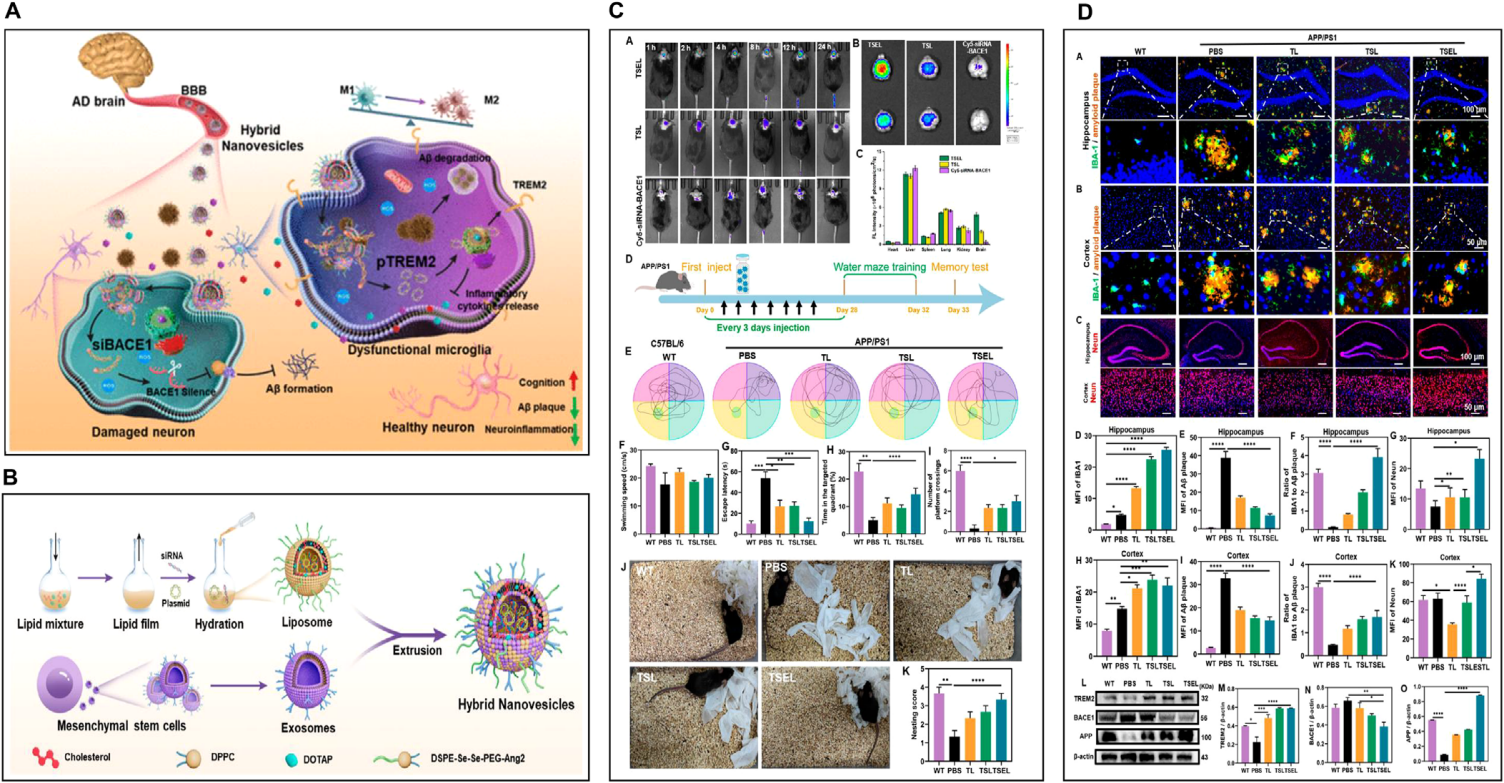
Biomimetic nanovesicles as a dual gene delivery system for the synergistic gene therapy of Alzheimer's disease. (A) Schematic illustration of the therapeutic mechanism of TSEL. (B) Schematic illustration of the synthesis process of exosome–liposome hybrid nanovesicles. (C) TSEL improves cognitive function in the APP/PS1 mouse model. (D) In vivo evaluation of multitarget therapy of AD mice by TSEL. Data were presented as mean ± SD (*n* = 3). *p < 0.05, **p < 0.01, ***p < 0.001. Reproduced with permission [199]. 2024 American Chemical Society.

There is already evidence of endothelial degeneration and weakened BBB function in AD, PD, and ALS, which also highlights the serious consequences of neurodegenerative diseases and neurovascular dysfunction in aging [196]. The occurrence of BBB dysfunction is accompanied by dysfunction of neurovascular system and loss of neuronal function as well as neuro‐inflammation [197]. In particular, neurovascular damage is also seen in both human and animal tissues with chronic psychological disorders [198, 199, 200]. In such a state, changes occur in the perivascular microenvironment [201], manifested by thickening of the basement membrane [202], astrocyte end‐foot deformation [203] (Figure 7), microglial activation [204], and chronic neuroinflammation. Secondary pathological changes develop with changes in the BBB microenvironment, and drug entry into the CNS is complicated by the onset of secondary pathology [205]. Studies have shown that downregulated expression of tight junction protein leads to space dilation around blood vessels and accumulation of toxic proteins, such as fibrinogen [206]. Vascular dysfunction also affects the cellular components of the neurovascular niche (e.g., pericytes) [207]. In fact, pericytes play a key role in BB integrity, and as blood vessel function declines, amyloid and proteoglycan deposits increase [208], so material formulas designed to deliver drugs across the healthy BBB face a number of obstacles that affect delivery to the diseased CNS [209, 210].

Besides, the role of exercise as a “noninvasive” intervention method in the prevention and treatment of neurodegenerative diseases has been widely recognized. Although the impact of exercise on OPCs is not specific to any particular pathway, this is very worthwhile and offers various advantages. Moreover, new and original sport assignments, for instance, skilled hand stretching tasks or complex running wheels, have already been suggested to promote the proliferation, migration, and differentiation of OPCs in animals, resulting in the generation of OLs and myelin regeneration. Meanwhile, exercises also have ability in the regulation of irisin release in muscles. And then, Irisin can regulate the release of BDNF, enhances the ability of synaptic mitochondria to transport glucose, and promotes the coupling efficiency between movement and respiration. In addition, exercise regulates mitochondrial regeneration and this affects mitochondria numbers. To be important, exercise is crucial for keeping the kinetic equilibrium of mitochondrial fission and fusion, as well as the structural integrity of mitochondria. Thus, the OPCs regulation by exercise can be fulfilled by ameliorating the brain microenvironment.

As we already known, OLs are myelin forming cells in the CNS, which are subjected to cellular stress and subsequent death in many neurodegenerative diseases. It is worth noting that live OLs and intact myelin sheaths are essential for the health of neurons. Since the abnormal WM and GM demyelination in patients with CNS diseases. The pathological evidence prove the clearly injury/destruction of myelin and OLs. Although there are currently several treatments available for inhibiting inflammatory by immune cells from blood in patients. However, there are limited treatments for improving OL pathology and enhancing neuronal health. In order to develop this new therapy, the deeply knowing of the developmental process of OLs is needed, Additionally, new tools for precise managing of neuronal and functional defects in animal studies. Obviously, by addressing known of the pathophysiological of OLs, the relationship between OLs and immune cells, and new models, we can better study the pathophysiological process of OLs.

### 3.4 In Cerebrovascular Diseases Studies

A stroke is a vascular brain injury caused by ischemia and/or bleeding that has a high lifetime risk. There are currently about 14 million new cases of stroke worldwide each year, of which 70% are ischemic and 30% are hemorrhagic [211]. Treated with tissue plasminogen activators (tPA) has advantages on terms of survival and function [212]. Obviously, tPA can promote the plasminogen conversion and the dissolution of clots [213]. However, tPA must be administered intravenously and the time window for intervention is narrow, with only 4–6 h after stroke recommended [214]. However, due to the need for systemic administration, which requires a high dose (0.9 mg/kg) intravenously, patients may suffer serious complications of intracranial and/or subarachnoid hemorrhage. Indeed, controlled release systems are urgently needed to achieve functional therapeutic benefits after stroke while reducing the risk of bleeding [215].

In addition, endovascular thrombectomy has been suggested for patients whose clinical severity does not match the infarct volume within 24 h of stroke [216]. Endovascular pathways also provide potential pathways for drug delivery systems to facilitate stroke recovery. In addition to this, it also provides an opportunity for controlled drug release for sub‐acute and chronic stroke, for example, to promote nerve regeneration or neuroplasticity [217]. The regenerative capacity of the CNS is limited due to a variety of biological processes [218], including the formation of glial scars. Chondroitin sulfate proteoglycan is a critical component of glial scars, and local delivery of chondroitin enzyme ABC can help address this obstacle [219]. Matrix metalloproteinases can help reshape the ECM and have potential therapeutic value [220]. Other strategies include neuroprotection, the delivery of growth factors through slow‐release materials to promote stem cell survival and differentiation [221, 222].

When we think of OL pathology, the first pathological feature that comes to mind is the injury/destruction of OL myelin units. Meanwhile, some diseases may also be related to the pathological process of OL. First, these are WM malnutrition and degeneration, such as heterochromatic leukodystrophy. In addition, as we discussed earlier, the regeneration of OLs seems to be critical in many neurological diseases, such as SCI, AD, or stroke, where myelin‐related processes are repeatedly disrupted in several cell types, indicating that myelin formation plays an important role in stroke, where OPCs are believed to promote angiogenesis. In addition, recent studies have shown that OL lineage cells could transform into antigen‐presenting cells in specific situation, indicating that OLs could be as immunomodulators [223]. Microglia are innate immune cells of the CNS. Their activation represents early events of disease development, loss of BBB function, and inflammation. The release of multiple factors has a negative impact on OLs and myelin. Recent studies have reported that OLs located in the CNS, also known as myelin forming cells, play a critical role in the early stages of myelin regeneration in stroke and temporarily alleviating symptoms of stroke. The clinical symptoms of stroke vary depending on CNS damage and the progressive reduction of nerve function due to the disruption of nerve transmission. The consequences are manifested as long‐term physical and psychological impairments as well as cognitive impairment [224], which is associated with pain and sleep disorders [225]. Additionally, Deussing et al. explored a new class of OPC subpopulations expressing corticotropin‐releasing hormone (CRH). Stroke and other cerebrovascular diseases induced brain injury rapidly induces OPCs to briefly express CRH. These cells gather around the injury site and exhibit a stronger ability to differentiate into myelin‐forming OLs. The target cells are OPCs expressing CRH receptor type 1 (CRHR1), and the differentiation rate of these cells is relatively slow. On the other side, after acute cerebral hemorrhage, although inhibiting the CRH/CRHR1 system can accelerate the generation rate of OLs, it will harm their long‐term survival. Under noninvasive conditions, the loss of function of the CRH/CRHR1 system can lead to an increase in OL generation after early birth and cause changes in myelin formation in adulthood. In summary, the researchers found that CRH derived from OPC not only actively affects the injury environment by interacting with OPCs expressing CRHR1, but also revealed that the G protein‐coupled receptor CRHR1 is a key regulatory factor for OL generation after early birth and has a lasting impact on myelin formation in adulthood. Here, the authors have revealed a brand‐new mechanism of neuro–OPC interaction: under pathological conditions such as brain injury, OPC can secrete the neuropeptide and regulate its differentiation rhythm and myelin formation by acting on specific OPC subgroups expressing this neuropeptide–CRH and this related signaling pathway is not only activated during acute injury and demyelination, but also plays a “molecular brake” role in early postnatal development, preventing OLs from maturing prematurely [226]. These findings for the first time demonstrate that OPCs possess neuropeptide secretion functions similar to those of neurons and that the neuropeptide–CRH signals can dynamically regulate myelin plasticity. This not only overturns the traditional understanding of glial cell functions but also provides a brand‐new intervention target and theoretical basis for the repair of cerebrovascular diseases and the treatment of demyelinating diseases such as MS. Similarly, photobiomodulation (PBM) with low‐level laser application has been proven to stimulate OPC proliferation, promote myelin repair, thereby enhancing neural plasticity and facilitating disease recovery, and has beneficial effects on cerebrovascular diseases. Unfortunately, there is currently no evidence that PBM therapy can specifically target OPCs. Since PBM has already been generally applied for enhancing the mitochondrial cytochrome *c* oxidase activity, reducing oxidative damage, and improving the activity of antioxidant enzymes. Thus, the protection of mitochondria; furthermore, by regulating OPC differentiation and myelin formation, improving the microenvironment, and promoting the rehabilitation of cerebrovascular diseases. However, the exact target of PBM still needs further research.

## 4 Conclusion and Prospects

In conclusion, OPCs have initial description as NSCs to generate OLs and play a crucial role in the CNS, participating in myelin formation, intercellular signal transduction, phagocytosis, and the formation and repair of the BBB. However, under pathological conditions, the microenvironment in the brain deteriorates, leading to changes in OPC‐related activities. The migration and proliferation of OPCs are impaired, which affects the differentiation of OPCs into mature OLs. Interruption of OPC differentiation can lead to pathological changes in myelin degeneration and neurodegenerative diseases. When it comes to OPCs, most people immediately think of their “main job” of developing into OLs, which wrap around nerve axons to form myelin sheaths, just like insulating wires, ensuring the efficient transmission of nerve signals [227]. In addition, it can have “precise conversations” with various types of cells such as neurons, astrocytes, and microglia, playing a crucial role far beyond imagination in brain development, homeostasis maintenance, and disease repair [228]. Indeed, we still have some unknown information and detailed molecular mechanisms participated in myelination and remyelination processes. The better understanding of myelin formation can be achieved to promote myelin regeneration, which is essential for treating demyelinating diseases such as MS. Moreover, It will be an interesting question whether OLs actively participate in the MS progression through persistent inflammation. Collectively, understanding the “relationships” between different types of neural cells is more important than understanding the cells themselves [229]. Recently, a research team in China has discovered that OPCs promote the exocytosis of neuronal lysosomes by contacting the neuronal cell body through processes and relying on neuronal activity. When the number of OPCs decreases or their branches are reduced, this reduction in contact leads to the accumulation of lysosomes, which in turn causes metabolic disorders in neurons and accelerates aging [230]. Similar phenomena have also been verified based on animal models. This founds provide new ideas for revealing the role of OPCs in neurodegenerative diseases, especially having potential application value in the prevention and treatment of age‐related lesions [231]. Future research can further explore the communication mechanism between OPCs and neurons, reveal their functional changes in both healthy and diseased states, and provide new targets and strategies for the treatment of neurodegenerative diseases [232].

Due to the BBB, delivering drugs to the CNS remains challenging in technical and clinical aspects. Advances in delivery systems to brain has broadened our knowledge and neurodegenerative diseases, psychiatric disorders, brain tumors, and brain injuries, from controlled, responsive, and tailored drug delivery systems. However, considering the huge challenges of testing engineered materials using rodent models, which can only partially simulate the biological barriers faced by human delivery system, making the actual application of these materials in human disease treatment some way off. In addition, although there are materials that can be delivered across the BBB, the delivery efficiency is still very low, and new material formulations are needed to increase breakthrough BBB delivery and distal target tissue penetration to promote cell repair. For instance, dynamic materials that are bio‐stimulating or environmentally responsive can overcome a range of obstacles and facilitate systemic delivery of the pathological CNS.

In addition, among many neurodegenerative diseases, the aging of OPC can lead to demyelination. Given the significance of OPC, target the aging and function of OPC. Strategies for improving OPC aging, restoring OPC function, enhancing the myelin repair process, promoting OPC differentiation, promoting myelin regeneration, and restoring neural circuits show great potential. In addition, combining strategies aiming to OPCs or its related microenvironments may supply synergistic effects and improve treatment outcomes. Thus, further research should be needed to intensify the understanding of the complex mechanisms of OPC‐related activities in the pathological environment. Revealing specific signaling pathways, the molecules involved, and the multiple of cellular interactions involved will lay the foundation for more precise and effective therapeutic interventions.

In parallel, the rapidly growing CNS drug toolkit is bound to improve treatment options for patients. Intrathecal pathways have been shown to be useful for CNS delivery of DNA, siRNA, or nanoparticles. Noninvasive approaches to the brain through systemic administration, especially for biologic drugs, will transform the treatment of neurodegenerative diseases and metastatic brain cancer that require repeated administration. In recent years, substantial progress has been made in both antibody and nanoparticle engineering and focused ultrasound mediated delivery. However, it is also necessary to further improve the efficiency of delivery to the CNS to avoid the deterioration of disease pathology.

New technical strategies allow greater access to target sites for local delivery approaches, such as electrode implantation for PD, local implantation with stereotactic devices for ALS patients. Therefore, the drug formulation of a local administration strategy is combined with systematic dosing to further improve outcomes. Meanwhile, seeking the potential of the emerging technologies, for instance, gene editing is expected to leverage the regenerative capacity of OPCs to promote myelin repair. Given that some cognitive dysfunction induced by neurodegenerative diseases is often closely related to the functional synapses deficits, it is critical to determine the possible role of OPCs in synaptic phagocytosis, which may explain the basis of the neurophysiological defects observed in some neurodegenerative diseases. Clearly, OPCs and OL‐related basic mechanism studies and nanotherapeutic technologies could provide multiple strategies for treatment of CNS diseases with personalized nanomedicine in future.

## Author Contributions

QX and XL designed the manuscript; wrote and revised the draft; data collection, analysis, and interpretation by QX, RS, YH, and XL. QX, RS, YH, LL, JT, XD‐L, and XL revised the draft. All of the authors approved the final version of the manuscript.

## Funding

The work was supported by the Natural Science Foundation of Hunan Province (No.2025JJ70598), The Science and Technology Innovation Program of Hunan Province (No.2022RC1231), The China Scholarship Council (CSC) for QX, and The Innovation Project of Postgraduate Research of Hunan Province (LXBZZ2024308) for RS and The Key Project of the Education Department of Hunan Province (No.25A0402).

## Conflicts of Interest

All of the author has no conflicts of interest to declare.

## Ethics Approval

The authors have nothing to report.

## Data Availability

All data generated or analyzed are included in this article.
